# Probiotic treatment in an intensive care unit: a narrative review

**DOI:** 10.1186/s40560-025-00803-0

**Published:** 2025-06-05

**Authors:** Sato Takeaki

**Affiliations:** Tohoku University Hospital Emergency Center, Seiryo-Cho 1-1, Sendai, Miyagi 980-0872 Japan

**Keywords:** Probiotics, Critically ill patients, Diarrhea

## Abstract

Diarrhea is common in critically ill patients and can lead to malnutrition, electrolyte imbalance, and dehydration. While its direct impact on outcomes, such as mortality or intensive care unit (ICU) stay, remains unclear due to inconsistent definitions, it often results from drug-induced causes, such as antibiotics and antacids. These agents can also contribute to dysbiosis and increase the risk of infections including *Clostridioides difficile* infections (CDI) and ventilator-associated pneumonia (VAP).

Probiotics, defined as live beneficial microorganisms, can counteract dysbiosis by modulating immune responses, restoring microbial balance, and reducing intestinal inflammation. Evidence suggests that probiotics may help prevent diarrhea and secondary infections. Clinical trials and meta-analyses have shown that probiotics may reduce the incidence of VAP, length of ICU stay, duration of mechanical ventilation, and potential in-hospital mortality in critically ill patients.

However, evaluating probiotic efficacy remains challenging due to the lack of standardized markers and the influence of confounding factors like antacid use. In a randomized controlled trial, synbiotic therapy was associated with improved fecal microbiota and reduced infections; however, the role of antacids was not addressed.

Probiotics are generally safe, although rare adverse events, such as probiotic-associated bacteremia, have been reported, particularly in immunocompromised individuals.

The 2024 Japanese Critical Care Nutrition Guidelines included a systematic review and meta-analysis supporting the potential benefits of probiotics in critically ill patients. However, due to significant heterogeneity in strains, dosing, duration, and concurrent antibiotic/antacid use, a weak recommendation (GRADE 2C; low certainty) was issued.

Future research should focus on the standardized evaluation of diarrhea and microbiota changes, the use of objective markers—such as fecal pH and short-chain fatty acid levels—and clarification of the interactions of probiotics with other medications. Comprehensive bowel management, including the cautious use of antibiotics and antacids, may be essential to fully recognize the therapeutic potential of probiotics in critical care settings.

## Clinical impacts of diarrhea, especially in the critical settings

Diarrhea can result in malabsorption, malnutrition, electrolyte imbalance, and dehydration. It is also a common symptom of infections, including antibiotic-associated enteritis such as *Clostridioides difficile* infection (CDI).

A systematic review (SR) of the epidemiology of diarrhea in critically ill patients found no significant impact on the duration of mechanical ventilation, intensive care unit (ICU) stay, or mortality [1]. However, because of the wide range of definitions and the reported incidence varying from 3.3 to 78%, the data should be interpreted with caution.

Diarrhea has multiple causes, with non-infectious factors, particularly drug-induced factors, being the most predominant. Common culprits include antibiotics, laxatives, prokinetic agents, and antacids [2]. Among these, antacids are associated with an increased risk of ventilator-associated pneumonia (VAP) [3], diarrhea, and CDI [4].

### Expected role of probiotics

Probiotics are defined as “live microorganisms that have a beneficial effect on the host when taken in sufficient amounts” and are represented by *Lactobacillus, Bifidobacterium* spp., and butyric acid bacteria.

Probiotics can target both healthy and ill individuals, with preventive or therapeutic effects. The intended outcomes may include addressing the root causes of diseases, metabolic imbalances, or alleviating symptoms during disease onset or progression.

In healthy individuals, probiotic use primarily aims to maintain overall health and prevent diseases. Many studies on probiotic applications have reported positive outcomes of biomarkers relevant to human health [5].

This manuscript primarily focuses on probiotics, while referencing relevant studies involving synbiotics, a combination of probiotics and prebiotics. Particular emphasis was placed on the effectiveness of probiotics in preventing diarrhea associated with dysbiosis.

Dysbiosis is a state of disrupted gut microbiota balance commonly observed in critically ill patients treated with antibiotics, immunosuppressants, or radiation therapy, leading to a loss of microbial diversity and altered intestinal function. Innate and adaptive immunity control the colonization niche of intestinal microbiota through various mechanisms, including the production of antimicrobial peptides and IgA antibodies.

Dysbiotic microbiota may actively influence the colonization niche by altering the functions of innate and adaptive intestinal immunity [6]. This may lead to an influx of pathogenic bacteria into tissues (bacterial translocation), causing a systemic inflammatory response syndrome (SIRS) originating from the intestinal tract.

Probiotics are hypothesized to restore a healthy intestinal environment by reducing intestinal inflammation and modulating immune responses. They stimulate intestinal epithelial cells and immune functions, thereby promoting the re-establishment of a balanced microbiota [7]. In addition, ingestion or administration of substances that promote probiotic growth supports disease prevention and helps maintain microbial homeostasis [8]. These mechanisms may contribute to preventing secondary infections in critically ill patients.

Probiotics have demonstrated efficacy in a range of clinical conditions, including necrotizing enterocolitis, antibiotic-associated diarrhea, recurrent CDI, *Helicobacter pylori* infections, inflammatory bowel disease, intestinal tumors, urinary bladder cancers, female urogenital infections, and postsurgical infections [9]. In critically ill patients, a recent meta-analysis showed that probiotics may reduce the incidence of VAP, duration of mechanical ventilation, length of ICU stay, and in-hospital mortality [10].

Evaluating the effects of probiotics presents several challenges. First, both qualitative and quantitative assessments of gut microbiota are difficult to perform, and reliable methods for determining the effectiveness of probiotic administration are limited. Analyses of fecal samples from patients with SIRS have revealed a decrease in specific bacterial species and short-chain fatty acid levels, along with elevated fecal pH [11]. Probiotic administration has been linked to increased levels of beneficial bacteria, decreased fecal pH, and reduced infection rates [12].

In a randomized controlled trial (RCT) involving 72 patients with sepsis who required mechanical ventilation, researchers evaluated the effects of synbiotics, probiotics, and prebiotics on enteritis, fecal bacterial counts, and organic acid concentrations [13]. The intervention group exhibited increased levels of beneficial fecal bacteria and organic acids, along with a reduction in enteritis and VAP. The duration of antibiotic therapy was shorter in this group. However, the study did not address the influence of antacid use, which has been associated with an increased risk of both diarrhea and VAP.

Potential indicators of the effectiveness of probiotic administration include fecal bacterial culture, quantification of short-chain fatty acid levels, and a reduction in fecal pH. However, implementing these assessments in routine clinical practice may be challenging.

### Adverse events associated with probiotics

Probiotics are living biological agents that rarely lead to adverse events such as bacteremia [14]. In a single-center retrospective observational study, five (0.08%) patients with *Clostridium butyricum* bacteremia were identified among 6,576 positive bacterial blood cultures. All five patients had been receiving probiotic formulations containing *C. butyricum*. Most cases occurred in immunocompromised patients, and one patient died of non-occlusive mesenteric ischemia [15].

Although probiotic-associated bacteremia remains extremely rare, it is a potential risk factor, particularly in hospitalized or immunocompromised patients.

### Probiotics for critically ill patients

In critically ill patients, probiotics are expected to offer several benefits, including the prevention of VAP and healthcare-associated pneumonia (HAP), reduction in hospital stay duration and mechanical ventilation periods, and potentially lower mortality rates. A recent SR found low-certainty evidence that probiotic administration in this population reduced the incidence of VAP and HAP, shortened ICU and hospital stays, and decreased the duration of mechanical ventilation; however, no effect on mortality was observed [16]. During the perioperative period of elective abdominal surgeries, both probiotics and synbiotics have been associated with reduced postoperative infectious complications and shorter hospitalization, with no reported adverse events [17].

In addition, an SR of probiotic use in adults and children receiving antibiotics revealed a significant reduction in the incidence of CDI without an increase in adverse events [18].

CDI is a particularly serious condition that is often triggered by antibiotic-induced dysbiosis in ICU settings. Although treatments such as vancomycin, metronidazole, and fidaxomicin are available, they may further disrupt the gut microbiota and contribute to CDI recurrence.

Probiotics are among the most promising non-antibiotic strategies for managing dysbiosis and preventing CDI [19]. Currently, neither the ASPEN 2022 [20] nor the ESPEN 2019 [21] guidelines include recommendations for probiotic use in clinical practice.

### Summary of probiotics in the Japanese Critical Care Nutrition Guideline 2024

The Japanese Critical Care Nutrition Guidelines 2024 [22] included an SR and meta-analysis indicating that probiotic treatment for critically ill patients may reduce in-hospital mortality, duration of mechanical ventilation, and incidence of infectious complications. In this SR, considerable variability was observed in probiotic interventions, including the types of strains used, dosages, and duration of administration (Table 1). Furthermore, no standardized indicators have been established to assess their efficacy. Notably, the cited studies did not investigate the differences between probiotic strains; therefore, the strain-specific effects remain unclear. Although the administered dosages were deemed sufficient, the duration of use varied widely.

**Table 1:** Summary of 16 randomized controlled trials included in the meta-analysis

No	Author	Population	Area	n	Kinds of probiotics	Probiotics administration	Primary outcome	Result	Diarrhea	Antibiotics	Antacid drug
1	Sharma B, et al. [23]	Pt with Acute pancreatitis	Asia	50	*L. acidophilus*, *B. longus*, *B. bifidum*, and *B. infantalis*	≦14 days	Gut permeability and endotoxemia	NS	n.m	n.m	n.m
2	Zeng J, et al. [24]	MV pt	Asia	235	*Bacillus subtilis* and *E. faecalis*	≦14 days	VAP, and mortality	Eff	n.m	69.5(pro) and 71.8% (con)	97.5 (pro) vs. 99.1% (con)
3	Alberda C, et al. [25]	ICU pt	America	32	*L. casei*	≦14 days	AAD, CDI	Eff	12.5% (pro) vs. 31.3% (con)	100%	n.m
4	Arpudh MA, et al. [26]	MV pt	Asia	146	*L. rhamnosus*	≦14 days	VAP, and mortality	NS	n.m	n.m	n.m
5	Johnstone J, et al. [27]	MV pt	America	2600	*L. rhamnosus* GG	≦14 days	VAP, and mortality	NS	76.9 (pro) vs. 76.3% (con)	83.1 vs. 81.9%	n.m
6	Litton E, et al. [28]	ICU pt	Europa	220	*L. plantarum* 299v	> 14 days	DAOH60, nosocomial infections	NS	n.m	80.9 vs. 62%	71.8% (pro) vs. 69.4%(con)
7	Barraud D, et al. [29]	MV pt	Europa	167	*L. rhamnosus* GG	≦ 14 days	VAP, ICU stay	NS	55.2% (pro) vs. 52.5% (con)	28-day antibiotic-free days were 16.4(pro) vs. 17.1(con) days	n.m
8	Tan M, et al. [30]	TBI pt	Asia	52	*B. longum, L. bulgaricus, Str. thermophilus*	> 14 days	Th1/Th2 cytokine levels, nosocomial infections, and mortality	Eff	n.m	100%	76.9% (pro) vs. 73.1 (con) %
9	Mahmoodpoor A, et al. [31]	MV pt	Asia	100	*L., B., Str. spp.*	≦14 days	VAP, hospital/ICU stay, and bacterial colonization	NS	14.6% (pro) vs. 27.8%(con)	n.m	n.m
10	Anand P, et al. [32]	MV pt	Asia	120	*L. acidophilus*, *L. rhamnosus B. longum*, and *S. boulardii*	≦14 days	VAP, CDI, and mortality	Eff	0 (pro) vs. 8% (con)	n.m	n.m
11	Habib T, et al. [33]	MV pt with trauma	Africa	65	*L. delbrueckii* and *L. fermentum*	> 14 days	VAP	NS	n.m	n.m	n.m
12	Behrooz N, et al. [34]	Neurosurgical MV pt	Asia	150	*Lactocare* (strain not specified)	≦ 14 days	VAP	Eff	n.m	n.m	n.m
13	Forestier C, et al. [35]	ICU pt	Europa	208	*L. casei, L. rhamnosus*	> 14 days	Delay in Pseudomonas aeruginosa colonization	Eff	n.m	105/106 (pro) vs. 101/102 (con)	n.m
14	Tsilika M, et al. [36]	Trauma MV pt	Europa	112	*L. acidophilus LA-5, L. plantarum B. lactis BB-12* and* S. boulardii*	> 14 days	VAP and sepsis	Eff	3.8% (pro) vs. 0 (con)	100%	100%
15	Wang J, et al. [37]	Respiratory ICU pt	Asia	61	Endo plasmegnic	> 14 days	Mortality, hospital stay, and gastrointestinal effects	NS	24 (con) vs. 43% (pro)	16.5 days(pro) vs. 19 days (con)	50% (pro) vs. 61% (con)
16	Tzikos G, et al. [38]	Trauma pt	Europa	103	*L. acidophilus LA-5, L. plantarum B. lactis BB-12* and* S. boulardii*	> 14 days	SSI	Eff	n.m	n.m	n.m

PT: Patients; Eff.: Effective; NS: Not significant; MV: Mechanically ventilated; TBI: Traumatic brain injury; L.: *Lactobacillus*; B.: *Bifidobacterium*; E.: *Enterococcus*; Str: *Streptococcus*; S.: *Saccharomyces*; VAP: Ventilator-associated pneumonia incidence; pro: Probiotics group; con: control group; N.M.: Not mentioned; PPI: Proton-pump inhibitor; AAD: Antibiotic-associated diarrhea incidence; CDI: *Clostridioides difficile* infection incidence; DAOH60: Days alive and out of the hospital by day 60

In treatment settings, probiotics are used after the onset of diarrhea or CDI to alleviate symptoms and restore gut microbiota balance. In preventive settings, probiotics are administered upon ICU admission to high-risk patients to reduce the incidence of VAP and CDI. Among the 16 RCTs included in this review [23–38], 15 primarily focused on prevention, while only one trial in patients with acute pancreatitis examined probiotics for treatment purposes [23].

Among the 16 included RCTs, probiotics were administered for more than 14 days in seven studies [28, 30, 33, 35–38], of which four demonstrated beneficial effects [30, 35, 36, 38]. In contrast, among the nine studies with a shorter administration duration (≤ 14 days) [23–27, 29, 31, 32, 34], four showed positive outcomes [24, 25, 32, 34].

Only seven of the 16 RCTs [25, 27, 29, 31, 32, 36, 37] reported diarrhea as an outcome, with wide variations in definitions and incidence rates ranging from 0 to 68%. In two RCTs [27, 29], in which the incidence of diarrhea exceeded 50% in both the probiotic and control groups, no significant treatment effect was observed. Conversely, the remaining five studies [25, 31, 32, 36, 37], in which the incidence was below 50%, reported favorable effects of probiotics.

Descriptions of antacid agent use were available in five studies [24, 28, 30, 36, 37] with reported administration rates between 50 and 100%.

Substantial heterogeneity in probiotic strains, dosage, duration, and concomitant use of antibiotics or antacids limits both the consistency of the meta-analysis results and the generalizability of the findings, despite favorable outcomes in some studies. Therefore, the Japanese Critical Care Nutrition Guidelines 2024 issued a weak recommendation (GRADE 2C), reflecting promising but inconsistent results and the overall low certainty of evidence based on the GRADE criteria.

## Limitations and future perspectives

In this review, we focused primarily on the preventive effects of probiotics. However, these outcomes may have been confounded by the presence of diarrhea as a clinical factor. Although probiotics can suppress diarrhea in critically ill patients [39], their efficacy depends on comprehensive management strategies. A multifaceted strategy to avoid overuse of antibiotics and antacids, balance bowel function, and prophylactic use of probiotics, may contribute to reduced ICU stay and lower rates of CDI incidence and recurrence [40].

From a clinical standpoint, without comprehensive control of diarrhea, the full therapeutic potential of probiotics may not be realized. Therefore, future clinical studies should aim to reassess the efficacy of probiotics while standardizing the assessment of diarrhea incidence and adjusting for confounding factors, such as the use of antibiotics and antacid agents. In addition, objective markers, such as fecal pH, short-chain fatty acid levels, and microbiota composition, are expected to serve as useful indicators of probiotics effectiveness. The development of simple and clinically applicable methods to assess these indicators is essential for advancing research and practice in this field.

## Data Availability

No data sets were generated or analyzed during the current study.

## Abbreviations

CDI: *Clostridioides difficile* infection
ICU: Intensive care unit
SR: Systematic review
VAP: Ventilator-associated pneumonia
SIRS: Systemic inflammatory response syndrome
RCT: Randomized controlled trial
HAP: Healthcare-associated pneumonia
